# Individualism, Polarization and Recovery from the COVID-19 Crisis

**DOI:** 10.1007/s10272-020-0928-7

**Published:** 2020-12-01

**Authors:** Barry Eichengreen

**Affiliations:** Department of Economics, University of California, Berkeley, 30 Evans Hall, MC #3880, Berkeley, CA 94720-3880 USA

## Abstract

Political polarization, meaning sharp differences in the political ideologies and preferences of the partisans of different parties, implies that members of one party are more likely to dismiss the policies and recommendations of spokesmen and appointees of the other party on the grounds that those policies and recommendations are informed by value systems inimical to their own. In the US, this means that when spokesmen for one party endorse masks, members of the other party reject them instinctively and automatically.

As Intereconomics and CEPS hold their annual conference, the United States and Europe are experiencing another wave of COVID-19 cases. When analyzing the causes and consequences, it is important to be aware that the situation is continuing to change – that one is aiming at a moving target. Moreover, every national case has its distinctive characteristics, though I will argue that one can still discern some regularities, albeit with exceptions that, as they say, prove the rule.

## Containment and recovery

A first regularity is that success at containing the spread of the virus and economic recovery go together. In a National Bureau of Economic Research working paper issued in October, Fernánde-Villaverde and Jones (2020) analyze data through the second quarter of the year. They situate different countries and US states in a two-by-two matrix, with COVID-19 deaths (high or low) on one axis and GDP loss (high or low) on the other. They show that most countries fall along the diagonal – high for both or low for both. This illustrates the well-known point that without successful containment of the virus, there can be no sustained economic recovery. Without containment, consumers will be reluctant to go shopping. Families will be reluctant to eat out. Businesses will be reluctant to invest given this subdued consumer spending and virus-related uncertainty.

There are exceptions, to be sure. Compared to other countries and US states, California has relatively low COVID-19 deaths but high GDP losses. This may reflect the idiosyncratic preferences of California's leaders and their constituents, Californians being notoriously idiosyncratic. More seriously, California is the bluest of blue, or Democratic-leaning, states and blue states approach the problem differently from red, or Republican-leaning, states (more on partisanship below). Or it may be that California's real-estate and high-tech heavy economy has been disproportionately affected by the pandemic.

Another exception is that the US economy recovered well in the second and third quarters of 2020 despite the country's poor performance on the public-health front. My suspicion is that this anomaly will resolve itself through weaker consumer spending going forward. The US hit a fiscal cliff in the third quarter, with the expiry of significant portions of the Coronavirus Aid, Relief, and Economic Security (CARES) Act. Substantial parts of the country that had avoided the virus have now become COVID-19 hotspots. There is a considerable likelihood of the US consumer now catching up to reality.

## The African exception

Sub-Saharan Africa, on the other hand, fits the usual pattern but in a surprising way. Many observers feared that COVID-19 would be a public-health calamity and economic disaster for the region, given its limited economic and financial resources and long-standing institutional weaknesses. Instead, COVID-19 cases and especially deaths have been relatively modest by international standards (Musa et al., 2020). In addition, the International Monetary Fund's October 2020 World Economic Outlook forecasts a 3% decline in real GDP in 2020, just half that of the advanced countries, and a full recovery in 2021 (IMF, 2020).

Why the region has done better than expected on both dimensions is not clear. Its youthful age structure helps with virus control, given what we know about the susceptibility of different age groups. Less certain is whether past experience with epidemics also helps. Public health services have experience dealing with other viruses, from which they and the public can learn. At the same time, my own research (Askoy et al., 2020a) shows that past epidemic exposure reduces trust in government, especially among individuals in their impressionable years (ages 18 to 25) when exposed to an epidemic, and that this decline in trust is very persistent, essentially permanent. The implications are not encouraging. When trust in the authorities is lacking, their advice is less likely to be taken. In particular, trust problems are apt to negatively affect compliance with lockdowns, mask mandates and vaccination. We document the point for vaccination in Askoy et al. (2020b).

## Individualism versus communitarianism

No single factor can account for why containment efforts have been more successful in some places than others, of course. Every unhappy pandemic-stricken country is unhappy in its own way. But my perspective, informed by the experience of the United States, points to two factors as deserving more attention: first, the distinction between individualism and communitarianism; and, second, the extent of political polarization. Individualists insist that the decision of whether to wear a mask or receive a vaccination, for example, belongs to the individual and that there is no role for government mandates. As the Idaho Freedom Foundation (Hoffman, 2020) put it in a post on 19 July, "The decision to wear a mask should be yours alone." A mandate, this author goes on, "denies me my right to make my own decisions based on conditions and desires unique to my situation."

Communitarians, in contrast, recognize that collective welfare depends on the behavior of all members of society. One person's decision to wear a mask or not may have first order implications for the health of others and for the community as a whole, given the contagious nature of the disease. The decision of whether or not to wear a mask should be taken collectively, or mandated by the appropriate officials, with the welfare of the entire community in mind.

It might seem obvious that the second view should prevail in the presence of a highly contagious life-threatening virus, but it is not obvious to many Americans. The country has a tradition of rugged individualism, passed down through the generations by Hollywood Westerns and authors like Frederick Jackson Turner (1894). Bazzi et al. (2020) show that residents of US counties with more frontier experience (counties on the outer reaches of the country for a larger number of years) show more individualistic attitudes and greater opposition to redistribution even today. More generally, the country's history of slavery and racial division makes it more difficult for Americans to think of themselves as a single community; in turn, this helps to explain why the US provides fewer collective goods and services than other countries (Alesina et al., 1999). It is because Americans think of themselves as individuals rather than members of a community.

Other traditions lean more in the direction of communitarianism in that they attach more value to social solidarity and political virtue rather than privileging the individual. Whether Asian countries tend to be more communitarian because of the influence of classical Confucian thought is disputed (see Fox, 1977). Be that as it may, casual empiricism suggests that more communitarian societies were more successful initially at controlling the spread of COVID-19 and that this translated into relatively good economic performance. But there are exceptions: New Zealand and Canada have done relatively well at flattening the curve while at the same time ranking relatively high in terms of individualism, except in comparison with the United States. Perhaps their success reflects other factors that swamp the negative impact of individualism. New Zealand is an island. It is led by a woman. This question deserves more systematic study.

## Polarization and partisanship

A second point is that the extent of political polarization also mediates the success, or otherwise, of COVID-19 containment measures, in turn shaping economic outcomes. This argument is similarly informed by the experience of my country, where political polarization as measured by Poole and Rosenthal (2007) and updated by inter alia Bonica et al. (2015) has been rising for years. Political polarization, meaning sharp differences in the political ideologies and preferences of the partisans of different parties, implies that members of one party are more likely to dismiss the policies and recommendations of spokesmen and appointees of the other party on the grounds that those policies and recommendations are informed by value systems inimical to their own. In the US, this means that when spokesmen for one party endorse masks, members of the other party reject them instinctively and automatically. One party becomes the party of draconian measures, the other the party of lax measures, leaving little room for compromise on middle-of-the-road policies.

Thus, Makridis and Rothwell (2020) have analyzed the responses of some 45,000 Americans to COVID-19 related questions. They find that views of the threat posed by the virus and of the advisability of non-pharmaceutical interventions are driven more by party affiliation than by the demographic profile of the individual (age for example), number of infections in the local area, and actual statewide policies. The article shows that insofar as these partisan affiliations translate into actual policies, they are associated with very real consequences for public-health and economic outcomes. Allcott et al. (2020) similarly document differences between members of the two US political parties, with Republicans engaging in less social distancing, expressing less concern about personal COVID-19 risk, and showing a greater tendency to downplay the future severity of the pandemic.

Just why political polarization has been increasing in the United States over time, to an even greater extent than in other countries, is another disputed question. My own view would emphasize the rise of gerrymandering in Congressional elections and the decline of party discipline in the Congress. Gerrymandering – the redrawing of Congressional borders to create “safe districts” for incumbents – reduces the incentive to appeal to the median voter in general elections. Rather, candidates of the favored party square off in primary elections, where the only relevant constituency are party members and the incentive is to adopt a more extreme position so as to appeal to that base (Mann, 2006). The explanation citing declining party discipline (Canen et al., 2020) suggests that the declining ability of party leaders to control their members has effectively limited scope for compromise. In contrast, the other commonly cited factor, namely the proliferation of specialised cable news channels and Internet-based publications, which create an echo effect, reinforcing more extreme preconceived positions (Prior, 2007), is common to the United States and other countries, and therefore cannot obviously explain the unusually sharp rise in polarization here.

## Conclusion

In thinking about economic recovery from the COVID-19 crisis, I have argued that recovery starts with controlling the virus. And virus control in turn starts with the politics. For better or worse, the personal and social attitudes informing those politics are deeply embedded in history. Past experience with epidemics – and also with recessions (Malmandier and Nadler, 2011; Giuliano and

Spillimbergo, 2014) – shape those attitudes and outlooks. In the US case, so too does the country's historic racial divide and its history as a frontier nation. History is not destiny, of course. Attitudes and outlooks, along with the politics they inform, can be altered by events, most powerfully by searing events that rise to the level of crises. We are about to see whether the COVID-19 crisis has such effects and whether the consequences are positive or negative.
